# Depression, Anxiety, Insomnia, and Quality of Life in a Representative Community Sample of Older Adults Living at Home

**DOI:** 10.3389/fpsyg.2022.811082

**Published:** 2022-04-01

**Authors:** Leif Edward Ottesen Kennair, Roger Hagen, Odin Hjemdal, Audun Havnen, Truls Ryum, Stian Solem

**Affiliations:** ^1^Department of Psychology, Norwegian University of Science and Technology, Trondheim, Norway; ^2^Department of Psychology, University of Oslo, Oslo, Norway; ^3^Modum Bad, Research Institute, Vikersund, Norway; ^4^St. Olavs Hospital, Trondheim, Norway

**Keywords:** anxiety, depression, insomnia, quality of life, mental health

## Abstract

**Background:**

The aim of the study was to explore symptoms of anxiety and depression, insomnia, and quality of life in a Norwegian community sample of older adults.

**Methods:**

A representative sample (*N* = 1069) was drawn from home-dwelling people of 60 years and above, living in a large municipality in Norway (Trondheim).

**Results:**

Based on established cut-off scores, 83.7% of the participants showed no symptoms of anxiety/depression, 12% had mild symptoms, 2.7% moderate symptoms, 1.5% showed severe symptoms of anxiety/depression. A total of 18.4% reported insomnia symptoms. Regarding health-related quality of life, few participants reported problems with self-care, but pain and discomfort were common (59%). Depression/anxiety, insomnia, and health-related quality of life showed moderate to strong associations.

**Discussion:**

The results suggest a close interplay between anxiety/depression, insomnia, and health-related quality of life in older adults.

## Introduction

Depression and anxiety are common psychological problems throughout the life span, and are typically comorbid (Byers et al., 2010). Anxiety and depressive disorders are in danger of being overlooked in the older population (Royal College of Psychiatrists, 2018), as other somatic conditions tend to become more pronounced with increasing age. Large-scale longitudinal epidemiological studies in Norway suggest an increasing prevalence of anxiety and depression in older age cohorts (Stordal et al., 2001; Solhaug et al., 2012). This is a case for concern, because such comorbidity is associated with poorer treatment outcome, cognitive impairments, and increased risk of suicide (Lenze et al., 2000; DeLuca et al., 2005).

The estimated point prevalence of major depressive disorder (MDD) for people above 65 years of age is 4–5% for women and about 2% for men in North American and European studies (Steffens et al., 2000; Sjöberg et al., 2017). The 12-month prevalence rates for MDD in older adults range from 0.3 to 10.2% in the United States, which is lower than common estimates of 10% in younger adults and 8–9% in middle-aged adults (Kessler et al., 2010). A recent study in the United Kingdom found no increase in prevalence of depression with higher age, however, but the study reported a marked increase in the use of antidepressant medication in older age groups (Arthur et al., 2020). Systematic reviews of anxiety disorders in older adults estimate the prevalence to be 1–15%, while the prevalence rate for anxiety symptoms is estimated to be significantly higher, between 15 and 52% (Bryant et al., 2008; Grenier et al., 2019).

Symptoms of anxiety and depression are also typically intertwined with insomnia, and there appears to be a bidirectional relationship between anxiety and depression with insomnia (Jansson-Fröjmark and Lindblom, 2008). Furthermore, reduced sleep quality is associated with increasing age, making insomnia more prevalent in older cohorts (Neubauer, 1999). Approximately 50–70% of all seniors report sleep difficulties (Fok et al., 2010; Jaussent et al., 2011; Smagula et al., 2016). Related research has also suggested that worry is a predictor of sleep problems (Pallesen et al., 2002), while a systematic review found female gender, depressed mood, and physical illness as risk factors for future sleep disturbances (Smagula et al., 2016).

It has been suggested that sleep quality acts as a mediator between depression and quality of life in older adults (Becker et al., 2018). Further, health problems and quality of life are associated with both anxiety and depression, and there might be an adverse effect of social isolation on the sleep quality of older adults (Yu et al., 2018). An important consequence of increased levels of symptoms such as anxiety, depression, insomnia, and reduced function in older cohorts, is an increased demand for health services with associated socioeconomic costs. More knowledge related to rates of such symptoms is therefore important when considering and designing interventions to reduce anxiety and depression among older adults. In this context, it is highly relevant to consider the interplay of these associated mental health problems.

There were several aims of this study. First, we wanted to investigate the self-reported symptoms of anxiety/depression and insomnia in a representative population of older adults living at home. Second, we wanted to explore health-related quality of life (including mobility, self-care, keeping up with one’s usual activities and pain/discomfort) in the sample. Third, we wished to investigate the associations between symptoms of anxiety and depression, and quality of life. Finally, the study set out to explore unique predictors of anxiety, depression, insomnia, and quality of life in older adults.

## Materials and Methods

### Participants and Procedure

The design was cross-sectional and used a representative sample of adults in Trondheim municipality aged 60 years and older, living at home. Trondheim has approximately 205.000 inhabitants, of which about 32.000 (16.8%) are aged 60 or above. The sample was drawn from the General Population Registry of Norway based on gender, age (60+), and area of residence (Trondheim Municipality).

Participants were invited to take part in a survey concerning anxiety, depression, quality of life, and sleep problems. There was no other specific inclusion- or exclusion criteria for participation. All individuals first received a postal invitation letter with information about the study and the questionnaires. A reminder was sent in the mail after two weeks to those who had not responded. We sent invitations to participate to 3,001 people and received 1069 (35.6%) responses.

The mean age of people not responding to the survey was 68.4 (*SD* = 5.6) compared to 68.9 (*SD* = 5.4) for responders. Regarding gender, 64.7% of women and 63.2% of men did not respond to the survey. No other information was available for people not responding to the survey.

The gender distribution in the sample that responded to the questionnaires was equal (50.2% women). Women reported more symptoms of anxiety/depression and insomnia than men, however, the effect sizes were small. Women also scored slightly lower on health-related quality of life (but not on the 0–100 VAS-scale). Mean age was 69.0 (*SD* = 5.3), and age was clustered in this distribution; 24.3% were between ages of 60–64, 28.2% were 65–69, 29.1% were 70–74, and 18.4% were 75 and older. Most of the sample were married/cohabitant (73%), 9.1% were divorced/separated, 8.5% were widows/widowers, 5.6% were single, and 3.5% had a romantic partner. Regarding education, 17% had completed compulsory school, 28% high school, 31% had a bachelor’s degree, and 24% had a master’s degree. With respect to work status, 19% were still working while 60% were retired. Ten percent had part-time work and 13% received social welfare benefits (note that the numbers do not add to a perfect 100% as some participants were both retired and engaged in part-time work). There were significantly more widows than widowers in the sample. In addition, more men reported to have completed higher education than women. A summary of the sample’s demographic characteristics is displayed in Table 1.

**TABLE 1:** Demographic characteristics of the sample (*N* = 1069).

	Total	Women	Men	*d*
Age	69.02 (5.43)	68.76 (5.57)	69.29 (5.27)	
Number of children	2.15 (1.05)	2.16 (1.03)	2.13 (1.08)	
PHQ-ADS	5.11 (6.68)	6.20 (7.68)	4.08 (5.39)*	0.32
ISI	5.01 (5.29)	5.50 (5.55)	4.58 (5.01)*	0.17
Quality of life—EQ	6.55 (2.18)	6.80 (2.42)	6.30 (1.89)*	0.23
EQ VAS (0-100)	77.95 (18.99)	77.46 (19.92)	78.43 (18.03)	
				*p*
Relationship status*	%			<0.001
Married/cohabitant	73.2	61.8	84.5	
Single	5.6	5.9	5.4	
Widow/widower	8.5	14.4	2.8	
Girlfriend/boyfriend	3.5	4.0	3.0	
Divorced/separated	9.1	14.0	4.3	
Education*				<0.001
Compulsory school	16.5	20.7	12.5	
High school	27.9	30.2	26.0	
University bachelor	30.8	33.1	29.0	
University master or more	24.0	16.0	32.4	
**Work status**				
Full-time work	19.3	15.8	22.7	
Part-time work	10.1	10.3	9.9	
Retired	60.0	59.4	60.5	
Social welfare	13.0	14.8	11.2	
Other	3.4	3.8	3.0	
**Treatment**				
Previously treated for anxiety/depression	17.8	23.7	12.1*	<0.001
Type of treatment				
GP	10.6	14.5	6.7	
Pharmacological	9.4	11.8	7.1	
Psychotherapy	10.8	13.3	8.2	
Admitted to hospital	3.0	3.9	2.0	
ECT	0.3	0.2	0.4	

Thirty-four participants were both retired and engaged in part-time work. GP, general practitioner; ECT, electroconvulsive therapy; PHQ-ADS, Patient Health Questionnaire Anxiety and Depression Scale; ISI, Insomnia Severity Index, *p < 0.01. Cohen’s d calculated using pooled standard deviations.

Ethical approval for the study was obtained from the Regional Ethical Committee for Medical Research (ref.nr. 2016/2265). Participants were anonymous for the research group, but invited participants were given contact information to the principal investigator if they had concerns related to the survey and the topics covered.

### Instruments

Different self-report instruments were used in this study and are described in detail below. Demographic variables such as sex, age, relationship status, living situation, number of children, level of education, work status, and whether one had received treatment for mental health-related problems were also collected. Self-reported treatment options were defined as: psychotherapy, GP, pharmacological, inpatient, and electroconvulsive therapy (ECT).

Symptoms of anxiety and depression were measured with the Patient Health Questionnaire Anxiety and Depression Scale (PHQ-ADS; Kroenke et al., 2016). The PHQ-ADS is a joint measure for symptoms of anxiety and depression, which is computed by summing the scores of the Patient Health Questionnaire-9 (PHQ-9; Kroenke et al., 2001) and the Generalized Anxiety Disorder 7 (GAD-7; Spitzer et al., 2006). The scores range from 0 to 48, with higher scores indicating more severe symptoms. Cut-off scores of 10, 20, and 30 are reported to correspond to mild, moderate, and severe levels of distress (depression/anxiety). The PHQ-ADS has proven to be a reliable and valid measure with satisfactory psychometric properties (Kroenke et al., 2016, 2019; Chilcot et al., 2018). Cronbach’s alpha for the PHQ-ADS in the current study was 0.95.

The Insomnia Severity Index (ISI; Morin et al., 2011) assesses symptoms of insomnia using seven items scored on a 0–4 scale. Cut-off scores can be interpreted as follows: absence of insomnia (0–7); sub-threshold insomnia (8–14); moderate insomnia (15–21); and severe insomnia (22–28). A cut-off score of 10 has been suggested as optimal for detecting insomnia in community samples (Morin et al., 2011). Cronbach’s alpha for ISI in the current study was 0.93.

The EuroQoL-5 Dimensions (EQ-5D-5L; Herdman et al., 2011) is a generic instrument commonly used for measuring health-related quality of life. The questionnaire can be used to compare health-related quality of life across persons with different disorders. The instrument is highly feasible with only five items (mobility, self-care, usual activities, pain or discomfort, and anxiety/depression). The EQ-5D-5L may be preferred to more extensive measures due to its brevity, easy administration, higher completion rates, sensitivity to change, and the ability to assess economical aspects (e.g., cost-effectiveness) of treatment (Holland et al., 2004). However, the measure could ignore relevant aspects such as existential or spiritual matters (Siette et al., 2021). The EQ-5D has been established as a feasible instrument in elderly, but most of the research has been conducted using the 3-point rating scale rather than the 5-point scale (Marten et al., 2021).

The five items are scored using a 1–5 scale (from no problems to extreme problems). Scores can be combined into a 5-digit code that describes the patient’s health state profile. The EQ-5D-5L also includes a visual analogue scale (VAS) which assesses health on a 0 (the worst health you can imagine) to 100 (the best health you can imagine) scale. Cronbach’s alpha for the five items in the current study was 0.79.

### Statistics

Descriptive statistics and cut-offs were used to describe symptoms of depression/anxiety, insomnia, and quality of life in the sample. Gender comparisons were conducted using *t*-tests and one-way ANOVAs. Correlation analyses were used to investigate the relationship between symptoms of depression/anxiety, insomnia, and health-related quality of life. Regression analyses explored unique predictors of health-related quality of life using the same variables. These analyses were conducted using SPSS version 28. Missing data was not imputed. Therefore, sample size for the regression analyses was 920. Finally, a SEM-analysis with PHQ-ADS and ISI predicting EQ-5D-5L was performed in Mplus version 8.6 (Muthén and Muthén, 2006). Full-information maximum likelihood was used with robust estimation (MLR) due to non-normality to make use of all available data. Model fit was evaluated with the following indices: Standardized Root Mean Square Residual (SRMR) (Browne and Cudeck, 1993) values less than 0.08 and values and Root Mean Square Error of Approximation (RMSEA) (Hu and Bentler, 1999) equal to or less than 0.06 indicate a good fit. For a Comparative Fit Index (CFI) and a non-Normed Fit index (NNFI; aka TLI) values greater than 0.95 indicate a good fit and values equal to or higher than 0.90 indicate an acceptable fit (Hu and Bentler, 1999).

## Results

### Symptoms

The mean score on PHQ-ADS was 5.11 and 5.00 on the ISI, indicating low levels of anxiety/depression, and insomnia for the sample in general. Women scored significantly higher than men on all symptom measures and quality of life, except for the 0–100 VAS of health-related quality of life. More women than men had received treatment for anxiety and depression, and 18% of the total sample had been treated for any kind of mental health-related problem. The most typical treatment was psychotherapy (11%) and GP (11%), followed by pharmacological treatment (9%), inpatient treatment (3%), and ECT (0.3%). The most common combination treatment was GP + pharmacotherapy (5.4%), followed by GP + psychotherapy (4.4%), while 2.8% had GP + pharmacotherapy + psychotherapy.

Cut-off values for anxiety/depression on the different instruments were used to explore rates in more detail, both for the total sample and for each gender separately. With respect to anxiety/depressive symptoms, 83.7% showed no symptoms of anxiety/depression, 12% had mild symptoms, 2.7% moderate symptoms, and 1.5 showed severe symptoms of anxiety/depression. Women showed more symptoms of anxiety/depression than men. For insomnia, 18.4% scored above the suggested cutoff, while 73.2% showed no signs of sleep problems. There were 21.5% of women scoring above cut-off compared to 15.6% of men. See Table 2 for more detailed information.

**TABLE 2:** Rates of distress, insomnia, and health-related quality of life in the sample.

	Anxiety/depression		
	
	Total	Men	Women
None (0–9)	83.7	88.2	79.0
Mild (10–19)	12.0	9.2	15.0
Moderate (20–29)	2.7	2.0	3.4
Severe (30+)	1.5	0.6	2.5
	**Insomnia**		
None (0–7)	73.2	76.9	69.1
Subthreshold (8–14)	19.1	17.0	21.5
Moderate (15–21)	6.8	5.4	8.4
Severe (22–28)	0.9	0.7	1.0
Cut-off 10	18.4	15.6	21.5
	**Quality of life (0**–**100)**		
0–24	1.7	1.3	2.1
25–49	4.9	4.4	5.4
50–74	21.1	20.4	21.8
75–100	72.3	73.9	70.7

*Numbers reported are percentages.*

### Quality of Life

Health-related quality of life scores (EQ-5D-5L) were examined according to the five dimensions to reveal how different aspects of quality of life and health were reported. Participants had few problems with mobility, however many experienced problems with pain and discomfort. Only 41.1% reported no problems with pain/discomfort. However, there were not that many with severe pain problems, as most reported only some problems with pain. Regarding anxiety and depression, 78.6% reported no such problems while 4.4% reported moderate to severe problems. The EQ-5D-5L profile was quite similar for men and women.

For the quality of life VAS scores, the majority (72.3%) reported a score of 75 or higher, while 1.7% reported very poor quality of life with scores of 24 or less. There was no significant gender difference on the VAS-scale, *t*(1038) = 0.83, *p* = 0.41. A summary of the EQ-5D-5L scores is displayed in Table 3.

**TABLE 3:** Quality of life among older adults.

	Mobility	Self-care	Usual activities	Pain/ discomfort	Anx/depr
**Total sample**					
No problems	82.8	95.7	85.1	41.1	78.6
Some problems	11.2	3.4	10.9	43.8	16.9
Moderate problems	3.7	0.6	2.7	11.1	3.4
Severe problems	2.1	0.3	1.1	3.2	0.9
Unable to/extreme	0.2	0.1	0.1	0.8	0.1
**Women**					
No problems	81.2	95.5	81.5	39.3	72.5
Some problems	11.7	3.0	13.2	42.1	22.1
Moderate problems	3.9	0.8	3.4	14.2	4.0
Severe problems	3.0	0.6	1.7	3.6	1.1
Unable to/extreme	0.2	0.2	0.2	0.8	0.2
**Men**					
No problems	84.5	95.9	88.8	42.9	84.6
Some problems	10.8	3.7	8.6	45.5	11.8
Moderate problems	3.4	0.4	2.1	8.0	2.8
Severe problems	1.1	0.0	0.6	2.8	0.7
Unable to/extreme	0.2	0.0	0.0	0.7	0.0

*Anx/depr, anxiety and depression. Numbers reported are percentages. Variables scored as “unable to” = unable to walk (mobility), unable to wash/dress (self-care), unable to do my usual activities (usual activities), extreme pain/discomfort (pain/discomfort), extremely anxious/depressed (anx/depr).*

The five digits EQ-5D-5L profiles were examined in order to reveal the most common health-related quality of life profiles. The most common profile for both men and women was a 1-1-1-1-1 profile, indicating no problems within any of the five categories. A summary of the most frequent EQ-5D-5L profiles is displayed in Table 4.

**TABLE 4:** Most frequent quality of life profiles.

EQ profile	Description	Total	Men	Women	60–64	65–69	70–74	75+
11111	No symptoms	36.3	40.6	31.9	38.6	35.6	36.2	34.4
11121	Some pain	27.5	31.1	23.9	25.1	27.8	28.6	28.7
11122	Some pain + some anxiety/depression	6.3	4.3	8.2	5.4	7.5	6.9	4.6
11131	Moderate pain	3.4	2.8	4.0	5.4	3.4	2.6	2.1
11112	Some anxiety/depression	3.1	0.9	5.4	3.5	3.1	3.3	2.6
21121	Some mobility + some pain	3.1	3.4	2.9	1.5	3.1	3.0	5.6
11132	Moderate pain + some anxiety/depression	1.4	0.8	2.1	0.8	2.0	1.6	1.0
11221	Usual activities + some pain	1.2	0.6	1.9	2.7	1.4	0.0	1.0
21221	Mobility + usual activities + pain	1.0	0.8	1.3	0.4	1.4	1.3	1.0

*Numbers reported are percentages. First digit, mobility; 2nd, self-care; 3rd, usual activities; 4th, pain/discomfort; 5th, anxiety/depression.*

### Relationship Between Anxiety, Depression, Insomnia, and Quality of Life

EQ-5D-5L consists of five items where the fifth item measures anxiety/depression. This particular item was therefore excluded from the correlational and regression analyses because it conceptually overlaps with other symptom measures used in this study. The correlations between the study variables ranged from non-significant to strong. Moderate to strong correlations were observed among symptom variables (PHQ-ADS and ISI), as well as between symptom measures and EQ-5D-5L. A summary of the correlational analyses is displayed in Table 5.

**TABLE 5:** Correlations between demographic variables, symptoms measures, and quality of life.

	1	2	3	4	5	6	7	8
1. Gender								
2. Age	0.06							
3. Widow/er	0.21**	0.18**						
4. Lives alone	0.24**	0.07*	0.49**					
5. Working	0.07*	0.54**	0.12**	0.08*				
6. EQ-5D-5L	0.08*	0.04	0.06*	0.11**	0.16**			
7. EQ-5D-5L VAS	0.01	0.04	0.08*	0.12**	0.14**	0.61**		
8. ISI	0.09**	0.02	0.06	0.12**	0.10**	0.48**	−0.48**	
9. PHQ-ADS	0.16**	–0.05	0.07*	0.11**	0.08*	0.55**	−0.49**	0.71**

*p < 0.05, **p < 0.01. PHQ-ADS, Patient Health Questionnaire Anxiety and Depression Scale; ISI, Insomnia Severity Index. EQ-5D-5L scores are without the fifth item, which assesses depression/anxiety. Spearman’s Rho reported for categorical variables.

Linear regression analyses used an enter method, with all predictors (age, gender, symptom measures, and socio-demographic variables) entered in the same step. In two independent analyses, the EQ-5D-5L total score (without the anxiety/depression item) and the VAS scale were entered as dependent variables. The total explained variance (adjusted *R* square) was 33% for EQ-5D-5L and 29% for the 0–100 VAS scale. When repeating the regression using all five items of the EQ-5D-5L, the amount of explained variance increased from 0.33 to 0.47. Symptoms of anxiety/depression and insomnia were significantly associated with health-related quality of life. Age and being a widow/widower did not predict quality of life, and there were no clear gender differences. However, having part/full time work was associated with better health-related quality of life. Multicollinearity was not a problem in these regression analyses, as VIF ranged from 1.1 to 2.1. A summary of the regression analyses is displayed in Table 6.

**TABLE 6:** Predictors of health-related quality of life.

	EQ-5D-5L* (adj. *R*^2^ = 0.33)			EQ-5D-5L VAS (0–100) (adj. *R*^2^ = 0.29)		
		
	β	*t*	*p*	β	*t*	*p*
Gender	–0.01	–0.37	0.714	0.06	2.04	0.042
Age	0.04	1.31	0.190	0.01	0.24	0.814
Widow/er	–0.01	–0.37	0.709	0.00	0.08	0.940
Lives alone	0.08	2.65	0.008	–0.08	–2.33	0.020
Working	–0.08	–2.45	0.015	0.08	2.40	0.017
ISI	0.25	6.58	<0.001	–0.30	–7.51	<0.001
PHQ-ADS	0.34	8.70	<0.001	–0.26	–6.35	<0.001

PHQ-ADS, Patient Health Questionnaire Anxiety and Depression Scale; ISI, Insomnia Severity Index. *EQ-5D-5L total score used without item 5 (anxiety/depression). Coding of dichotomous variables: Gender (Male = 1, Female = 2), Widow/er (Yes = 1, No = 0), Lives alone (Yes = 1, No = 0), Working (Yes part/full time = 1, No = 0).

**FIGURE 1:**
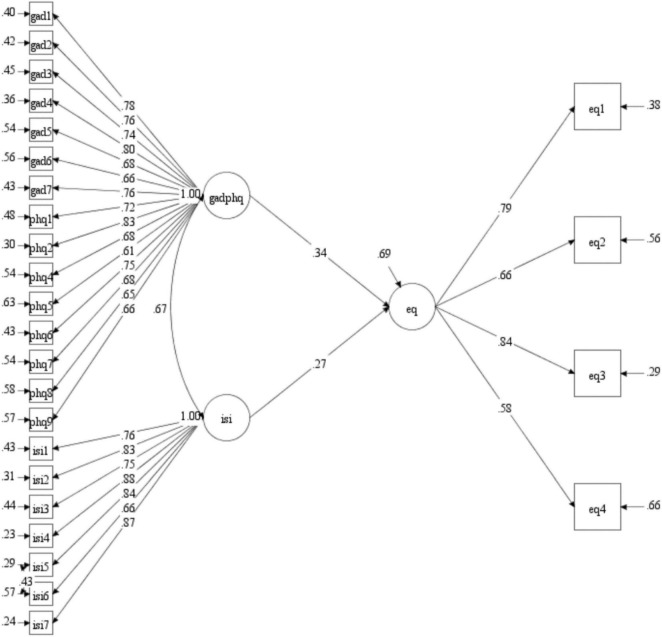
The SEM analysis with PHQ-ADS and ISI predicting health-related quality of life.

The fit for the initial SEM-model was not within acceptable range (*χ^2^* = 1674.723, *df* = 347, *p* < 0.001; RMSEA = 0.074; CFI = 0.832, TLI = 0.817), and modification indices indicated that two items were problematic. One item from the PHQ-9 measures sleep related problems and had strong residual variance with ISI. One item in the EQ-5D-5L measure assesses symptoms related to anxiety and depression and had strong residual variance with both GAD-7 and PHQ-9. These two items were taken out of the model. The revised model opened for residual correlation between ISI item 5 and 6. For the model in Figure 1, the results were acceptable, *χ^2^* = 883.127, *df* = 295, *p* < 0.001; RMSEA = 0.054; CFI = 0.916, TLI = 0.907. The results showed that levels of anxiety/depression and insomnia predict levels of subjective well-being in this elderly sample and explain 30.7% of the variance.

## Discussion

Regarding prevalence rates, the mean scores on the symptom measures suggested low levels of anxiety/depression and insomnia for the sample in general. In fact, the sample reported less symptoms of anxiety/depression compared to younger age groups from the same community (e.g., Solem et al., 2015). However, age was not a significant predictor of quality of life in the regression analyses. Whereas women scored consistently higher than men on all symptom measures used in this survey, female gender was not a stable predictor of health-related quality of life. Despite reporting overall low levels of symptoms, approximately one fifth of the sample had been in treatment for mental health problems. Insomnia, anxiety/depression, and health-related quality of life showed a close relationship to each other. Finally, the most common complaint reported in this sample of older adults living at home, was a somewhat reduced health-related quality of life due to pain/discomfort.

One of the aims of the study was to explore symptoms related to anxiety/depression and insomnia in a representative sample of older adults living at home. In our sample, 4.2% showed moderate to severe symptoms of anxiety/depression. Previous research has suggested a point prevalence of depression of 4.4% for women and 2.7% for men (Steffens et al., 2000). As for anxiety, Bryant et al. (2008) and Grenier et al. (2019) found prevalence rates ranging from 1 to 15% in their studies. Our results are thus in line with prevalence rates from other studies on older populations.

With respect to sleep disorders, studies have found that approximately 50% or more of seniors’ report sleep difficulties (Fok et al., 2010; Jaussent et al., 2011). In the current study, the figure for insomnia ranged from 7.7 to 18.4%, which is significantly lower. A possible explanation could be that other studies have investigated sleep problems in a broader sense, whereas our study focused on problems related specifically to insomnia. However, a Swedish study found that 13% of older adults without pain scored above 15 points on the ISI (Dragioti et al., 2017). In comparison, 7.6% in the current sample scored above this cutoff, again suggesting that symptoms of insomnia were quite low in our study sample.

Regarding health-related quality of life, few participants reported problems with self-care, but pain and discomfort were common (59% in the sample). It is important to note though, that this is a sample of older adults living at home, and there should be no surprise that self-care is not a major problem in such a population. The proportion of participants that reported perfect health (36%; with the 1-1-1-1-1 profile on the EQ-5D-5L) was comparable to rates from the general population in countries such as Poland (39%) and United States (35%) (Golicki and Niewada, 2017). The mean 0–100 score of 78 was also comparable to older age groups in Australia where scores ranged from 73 to 79 (McCaffrey et al., 2016), and close to that of Germany with scores from 80 to 90 (Hinz et al., 2014). Working part or full time was associated with better quality of life, which suggests that work may serve as a protective factor for health-related quality of life in the elderly population. Furthermore, the results showed that being a widow or widower was not associated with poorer health-related quality of life, whereas to live alone without a partner was a significant predictor of reduced quality of life. The protective factor of both work and living with a partner may possibly be explained by the fact that both work and living with someone serve as important parts of a persons’ social network, which in turn is a well-known contributor to improved health, both physically and mentally. Importantly, to have social relations may buffer against loneliness, which is associated with poorer quality of life in the elderly (Jakobsson and Hallberg, 2005).

The structural equation model showed that quality of life was predicted by anxiety/depression and sleep disturbances. The final model gained an acceptable fit to the data, explaining about 30% of the variance in quality of life. If the fifth item (anxiety and depression) of the EQ-5Q-5L was included, the explained variance increased to 47%. However, the fifth item was excluded from the main analysis to avoid extensive overlap between predictor variables and the EQ-5Q-5L. However, removing this item did not influence the significance of the predictor variables. The only difference observed was that gender became significantly associated (*p* = 0.042) with EQ-5Q-5L when the full scale was used.

In summary, the results suggest that anxiety/depression, insomnia, and quality of life are intertwined. Therefore, it is difficult to know what the primary or secondary problems are. The results from the SEM model supported the notion that comorbidity could decrease quality of life (Lenze et al., 2000; DeLuca et al., 2005). However, there were large amounts of unexplained variance in the model. Other potential predictor variables could have been included to increase explanatory power. It is likely that adding factors such as financial situation, somatic comorbidity, and social networks could have increased our understanding of the participants quality of life (Netuveli et al., 2006). When interpreting the results, it is important to keep in mind that the sample reported relatively low levels of symptoms, which may indicate that their health-related quality of life largely depend on matters not measured in this study. For example, even though the EQ-5D-5L is a well-established measure of health status in the elderly (Holland et al., 2004), this instrument does not capture personal circumstances, which may be of importance for the perceived quality of life in older adults (Siette et al., 2021).

Findings from the current study were not in perfect correspondence with those of a large epidemiological study from rural areas of Norway (Stordal et al., 2001). In contrast to our findings, they found that symptoms of depression *increase* with age and that there are no clear gender differences. There may be different reasons for these incompatible results such as study design (e.g., sample size and response rate), sample characteristics (e.g., urban vs. rural; living at home vs. general older adult population), and choice of measurements (PHQ-9 vs. the Hospital Anxiety and Depression Scale; HADS). Our study was representative of the older population in general except for lower participation among the 75+ group. It should also be noted that the HADS scale has been subject to serious criticism due to poor psychometric properties (e.g., Cosco et al., 2012; Coyne and van Sonderen, 2012a,b). In contrast, the current study used the PHQ-ADS to assess anxiety/depression, and the correlation between HADS and PHQ-9 is only low-moderate (Hansson et al., 2009). However, our results corroborate those of Arthur et al. (2020) who did not find an increase in depression prevalence rates with older age.

Regarding gender differences, our findings mirror statistics from the Norwegian Prescription Database and Statistics Norway in that women more frequently visit their GP for matters relating to mental health and are prescribed more antidepressants than men. This gender difference occurs irrespectively of age. Furthermore, the results speak to the complexity of mental health in older age and observed increases in anxiety/depressive symptoms could occur in the context of medical comorbidity rather than represent an independent effect of aging (Wu et al., 2012).

### Limitations and Suggestions for Future Research

The current study used a cross-sectional design, and we cannot conclude that any of the associations found between the variables considered in this study are causal. Thus, future longitudinal studies are warranted for considering any causal pathways between these constructs. The drawing of a representative sample is a strength of the current study, however, the response rate of 35% raises questions regarding eligible participants who chose not to take part in the study. People suffering from depression/anxiety or insomnia might be less interested in participating in research thus resulting in a selection bias. However, when comparing the samples with statistics collected from population-based registries, the sample seemed quite representative of the general older population with a gender distribution of 50.2% women. The group over 75 years was underrepresented. A major limitation relates to the use of only self-report measures, as no clinician-based diagnostic assessments were undertaken.

The current findings suggest that interventions are warranted to reduce suffering among older adults with anxiety/depressive symptoms. The close connection between insomnia, anxiety/depression, and health-related quality of life also needs to be investigated further. Correlations between study variables were moderate to strong, suggesting that there may be shared pathways. Therapeutic interventions that may reduce suffering in one area may therefore influence overall symptomatology. Transdiagnostic treatments that could address all these problems simultaneously would therefore be highly advantageous, since this would likely reduce the levels of distress and increase quality of life.

## Data Availability Statement

The raw data supporting the conclusions of this article will be made available by the authors, without undue reservation.

## Ethics Statement

The studies involving human participants were reviewed and approved by Regional Ethical Committee for Medical Research (Mid-Norway). The patients/participants provided their written informed consent to participate in this study.

## Author Contributions

LK, RH, OH, and SS collected the data in this survey. All authors have contributed equally in writing up the manuscript and approved the submitted version.

## Conflict of Interest

The authors declare that the research was conducted in the absence of any commercial or financial relationships that could be construed as a potential conflict of interest.

## Publisher’s Note

All claims expressed in this article are solely those of the authors and do not necessarily represent those of their affiliated organizations, or those of the publisher, the editors and the reviewers. Any product that may be evaluated in this article, or claim that may be made by its manufacturer, is not guaranteed or endorsed by the publisher.
